# The chemical constituents of *Sterculia comosa* (wall) Roxb woods for arginase inhibitory, antioxidant activity, and molecular docking against SARS CoV-2 protein

**DOI:** 10.1016/j.heliyon.2022.e08798

**Published:** 2022-01-19

**Authors:** Rini Prastiwi, Berna Elya, Muhammad Hanafi, Rani Sauriasari, Yesi Desmiaty, Ema Dewanti, Rina Herowati

**Affiliations:** a Faculty of Pharmacy and Science, Universitas Muhammadiyah Prof. Dr. Hamka, 1340, Jakarta, Indonesia; b Faculty of Pharmacy Universitas Indonesia, Depok, 16424, West Java, Indonesia; c Research Centre for Chemistry - National Research and Innovation Agency (BRIN), Indonesia; d Faculty of Pharmacy Universitas Pancasila, Jakarta, West Java, Indonesia; e Faculty of Pharmacy Universitas Setia Budi, Surakarta, Central of Java, Indonesia

**Keywords:** Arginase, Antioxidant, SARS CoV-2, *Sterculia comosa* (wall) Roxb., Molecular docking

## Abstract

Flavonoids and phenols have an arginase inhibitory and antioxidant activity. The *Sterculia* genus has phenols and flavonoids content. This study aimed to investigate the arginase inhibitory and antioxidant activity of the chemical constituent of *Sterculia comosa* (wall) Roxb and also their binding affinities to arginase. The most active extract was methanol extract. This active extract was determined for its arginase inhibitory and antioxidant activity, determined the total phenols and total flavonoids, and identified chemical compound. The methanol extract has IC_50_ 2.787 μg/ml for arginase inhibitory activity and IC_50_ 4,199 μg/ml for DPPH scavenging activity. The total phenols 723.61 mg GAE/gr, total flavonoids content 28.96 mg QE/gr extract. The chemical constituent: KC4.4.6 ((-)-2-(E)-caffeoyl-D-glyceric acid) and KC4.4.5.1 (trans-isoferulic acid) have an arginase inhibitory activity KC4.4.6: 98,03 μg/ml and KC4.4.5.1: 292,58 μg/ml. Antioxidant activity with DPPH methods KC4.4.6: 48,77 μg/ml and KC4.4.5.1: 88,08 μg/ml. Antioxidant by FRAP methods KC4.4.6: 16,4 FeEAC mol/g and KC4.4.5.1: 15,79 FeEAC mol/g. The isolate trans-isoferulic acid predicted has good interaction to arginase. Isolate KC4.4.6. Predicted has good interaction to PLPro of SARS CoV-2 PLpro. However, both isolates did not show good interaction to 3CLPro, nsp12, and Spike protein of SARS CoV-2.

## 1 Introduction

The rapid and progressive spread of the SARS-CoV-2 virus pandemic caused by the coronavirus (COVID-19) has severely affected thousands of people's health, the world's health system and has had a significant impact on the global economy. Characteristics of SARS-CoV-2 with influenza are the higher transmission rates and the greater risk of death from COVID-19, mainly due to Acute Respiratory Distress Syndrome (ARDS). The leading cause of death from COVID-19 in the elderly and those with compromised immune systems is respiratory failure. Several patients exhibited cardiovascular-related pathologies, including Congestive Heart Failure (CHF) and cardio-respiratory medullary cardiac dysfunction. Cardiovascular complications and focus of ACE2 as a co-receptor for SARS-CoV-2 are essential in this regard. This viral infection depends on the entry of the virus through the host to replicate. Coronaviruses such as SARS-CoV-2 and SARS-CoV-1 use the host protein angiotensin-converting enzyme-2 as a co-receptor to gain access to the lungs and brain (Cheng, 2020).

In coronavirus disease, the elderly and those with disorders such as hypertension, chronic obstructive pulmonary disease, diabetes, and cardiovascular disease are more likely to experience this virus adverse effects and become critical. They can rapidly develop acute respiratory distress syndrome, septic shock, metabolic acidosis, and coagulation dysfunction, possibly leading to death. Arginine supplementation can play a role in such a scenario, given the possibility of being in a state of deficiency (Mahmoud et al., 2020). It is known that the use of L arginine supplements reduces the length of time in hospital and reduces the use of respirators in COVID-19 patients (Giuseppe et al., 2021). Nitric Oxide (NO) is an essential molecule in regulating intercellular signaling and is involved in various processes, including regulating endothelial function. NO has antimicrobial activity, including against bacteria, protozoa, and some viruses. NO produced by an enzyme that catalyzes L-arginine oxidation to NO and L-citrulline, namely NOS (Nitric Oxide Synthase). Aubrey et al.'s (2005) research gave the result that NO specifically inhibited the SARS CoV-1 replication cycle, especially at the beginning. NO production by iNOS produces antiviral effects (Aubrey-chimene et al., 2005). In the COVID-19 pandemic, neonatal patients' treatment with inhaled NO was beneficial (Lu, 2020). Coronavirus and influenza are pandemic viruses that can cause lung injury and death due to ARDS. Viral infection can cause a "cytokine storm" that leads to pulmonary capillary endothelial cell activation, neutrophil infiltration, and increased oxidative stress. ARDS, characterized by severe hypoxemia, is usually accompanied by uncontrolled inflammation, oxidative injury, and damage to the alveolar-capillary barrier. Increased oxidative stress is a major cause of lung injury, including acute lung injury (ALI) and ARDS, two clinical manifestations of acute respiratory failure with high morbidity and mortality rates (Cheng, 2020). Efforts to find effective compounds in inhibiting infection by the coronavirus that causes severe acute respiratory syndrome (SARS-CoV-2) are still needed. Molecular docking analysis needed to find effective compounds against various target proteins for the treatment of coronavirus infection. This virus encodes a replicase complex (ORF1ab). They expressed in the form of the polyprotein (pp), which synthesizes nonstructural protein (nsp, nonstructural protein) and 4 structural proteins: spike protein (S), envelope (E), membrane (M), and nucleocapsid (N), during the proteolytic process. The main protease, 3CL protease (3CLpro), is a key enzyme in processing pp1a and pp1b polyproteins. ORF1a and ORF1b terminated by papain-like protease (PLpro, nsp3) and 3C-like protease (3CLpro, nsp5) to produce nsp. The 3CLpro protein has an important function and is considered an active target for antiviral drugs. PLpro is an enzyme indispensable for viral replication and infection and is an essential target for coronavirus inhibitors (Yu et al., 2020). Recent studies have shown that 2019-nCoV uses angiotensin-converting enzyme-2 as an entry receptor for entry into host cells. Protein S, a type 1 glycoprotein on the virus's surface, plays a vital role during virus entry into the host cell. Protein S helps the virus bind to host acceptors. This protein S from CoV2 has a strong affinity for human angiotensin-converting enzyme-2 (ACE-2). Compounds that can inhibit this protein will potentially inhibit the entry of the CoV-2 virus into the human body (Yu et al., 2020; Yulong et al., 2020; Zhu et al., 2020). the inhibitory effect on arginase enzymes such as methanol extract from *Scutellaria indica* and *piceatannol-3′-β-D-glucopyranoside* compounds from rhubarb extract (Kim et al., 2013). Several studies on the inhibition of arginase activity in some plants have resulted that (Najid et al., 2018; Minozzo et al., 2018; Girard-Thernier and Demougeot, 2016; Lim et al., 2012; Correa et al., 2013; Bordage et al., 2013). Some Sterculia plants also provide good arginase enzyme inhibitory activity such as Sterculia macrophylla Vent (Rini et al., 2018). Administration of antioxidants (vitamin C) shows reduce oxidative stress, which causes acute inflammation and lung injury. Antioxidants' administration also reduces the risk of viral infections and improves symptoms (Cheng, 2020). Sterculia has an antioxidant activity *Sterculia rubiginosa* Zoll. Ex Miq. (Rini, 2018, Rini, 2020a), *Sterculia stipulata* Korth (Rini *et al.*, 2020b).

## 2 Materials and methods

### 2.1 Materials

*Sterculia comosa* (Wall).Roxb. Plant woods collected from Botanical Garden of Bogor. This plant determined in Botany Herbarium Research Institute, Cibinong, West Java, Indonesia. Aqua bidestillata from local supplier. Arginase enzymes, substrate L-arginine, maleic acid, manganese sulfate from Sigma (Singapore). Urea assay kits from Quantichrom® Bioassay (United States), nor-NOHA (N^ω^-hydroxy-L-arginine) as a standard from Cayman (USA). Dimethyl sulfoxide (DMSO), methanol pro-analysis, ethyl acetate pro-analysis, n-hexane pro-analysis from Merck (Germany). The solvents used in the study were n-hexane, ethyl acetate, and methanol purchased from local suppliers. Some chemical reagents determine the content of total flavonoids, total phenols and antioxidant Activity by DPPH (2,2-Diphenyl-1-picrylhydrazyl) method. TPTZ (2,4,6)-Tris(2-pyridyl)-s-triazine from Merck (Germany) for antioxidant FRAP method. Six natural compounds, i.e. trans-isoferulic acid, KC4.4.6, dihydroxy-dimethoxyflavone, dihydroxy-trimethoxyflavone, piceatannol, as well as isoquercetin were used as tested ligands, while nor-NOHA used as active control. The protein used as the macromolecular target was arginase. The hardware used in this study was a desktop with Intel I7 @3,6 GHz, 8 Gb RAM. Installed software and web tool used were MarvinSketch 17.8, VegaZZ 3.1.2, AutoDock Tools 1.5.6, and PLIP (fully automated protein-ligand interaction profiler).

### 2.2 Extraction

The extraction was done by the maceration method using n-hexane, ethyl acetate, and methanol solvent. The extract dried in a vacuum of the rotary evaporator at temperature of 50 °C, continued in waterbath at 50 °C. The three extract tested the arginase inhibitor and antioxidant Activity. The active extract determine the flavonoids total and phenols total.

### 2.3 Arginase activity

For initial screening of the extract's inhibitory activity (the final concentration of the test sample in the reaction is 100 μg/ml). The concentration of stock made is 450 μg/ml. Ten (10) μl extracts (450 μg/ml) added 15 μl of arginase 1 U/ml enzyme solution and 20 μl of L-arginine 570 mM as substrate was added to each well, shake 5 s. The mixture was incubated for 30 min at 37 °C. Each sample has added to the mixture of reagents A and B (100 μl) from the urea kit, shake 5 s, and incubated at room temperature for 60 min. The result was observed with a microplate reader at 430 nm. The control used is the DMSO solution made with the same DMSO concentration used in the extract. The positive performed under the same conditions and nor-NOHA as a positive control. The experiments were performed in triplicate.

### 2.4 The antioxidant activity

The extracts (20 μg/ml) of sample in methanol were reacted with 180 ml of 150 μmol/l DPPH (2,2-diphenyl-1- picrylhydrazyl) in methanol solution at room temperature. For a control, methanol used to replace the sample. The incubated at room temperature for 40 min in the dark place. The absorbance was measured at 517 nm. The positive control was quercetine. The antioxidant capacity was calculated using the following:

(1) $$\text{Antioxidant ​activity}\left(\text{\%}\right)=\frac{Absorbance\;control\;-Absorbance\;sample}{Absorbance\;control}\times 100$$

The procedure according to Bobo Garcia (Bobo-garcía et al., 2015).

The FRAP method, for sample preparation, was carried out by five (5) mg sample (extract/quercetin) of 5 mg dissolved in 2 ml methanol p.a, (Concentration to 2500 μg/ml). The stock made different concentration for determined FeEAC. Piping 30 μl samples into the well. The sample dissolved in methanol. Then added 270 μl FRAP reagents Buffer: TPTZ: FeCl3.6H2O = 10: 1: 1) shaken and incubated at 37 °C for 30 min. The mixture read at a wavelength of 593 nm. As blank methanol was used to replace the sample, which contains a mixture of 30 μl methanol and 270 μl FRAP reagent. The plate blank contains methanol 300 μl. FRAP reagent = 10: 1: 1 reagent (buffer acetate: TPTZ: FeCl_3_.6H2O). The standard curve uses AFS. This method refers to the research of Pereira et al., (24) and Wong et al., (25).

(2) $$\text{FeEAC}=\frac{\Delta A}{GRAD}\times \frac{Av}{Spv}\times \text{D}\times \frac{1}{Cext}\times 10^{5}$$

The formula, FeEAC was the equality of ferric ions with antioxidant activity (μmol/g), which ΔA = absorbance of samples that have been reduced by blank, GRAD (M^−1^) was the gradient of the AFS calibration graph, Av = total volume for the test (300μl), Spv = sample volume (30μl), C_ext_ = concentration of sample stock, weight (gram) in volume (g/l), D = dilution factor for sample before analysis (D = 1 if sample was not diluted). GRAD (gradient) was determined from the calibration curve on AFS.

### 2.5 Determined the total phenols content (TPC)

TPC expressed as mg Gallic acid equivalents per gram of dried extract (mg GAE/g Extract). A total of 20 μl extract added with 100 μl of Folin-C Reagent (1:10), treated for 60 s, and then allowed to stand for 4 min. Added with 80 μl of the solution of 7.5% sodium carbonate (Na_2_CO_3_) in water, shake for 60 s. This mixture is incubated at room temperature in a dark place for 2 h. Read at 600 nm. The concentration of extract in the sample made at 100 μg/ml. The concentration of a stock solution made was 1000 μg/ml. Blangko was a sample replaced with methanol. The treatment was the same as the sample. Determining the total phenol content using gallic acid standards, total phenol was calculated as gallic acid's equivalence (mgGAE/gram). This method according to Farasat (Farasat & Khavari-nejad 2014).

### 2.6 Total flavonoids content (TFC)

The total flavonoids content determined by the method described by Farasat et al. with slight modification (Farasat & Khavari-nejad 2014). The extract (20 μl) in methanol was added to 20 μl of AlCl_3_.6 H_2_O 10% and 20 μl of 1 M potassium acetate and 180 μl of distilled water, and left at room temperature for 30 min. The solution mixed correctly, and the color intensity read at 415 nm after 15 min. Quercetine use as the standard. All experiments were done in triplicate.

### 2.7 Isolation

**Figure 1:**
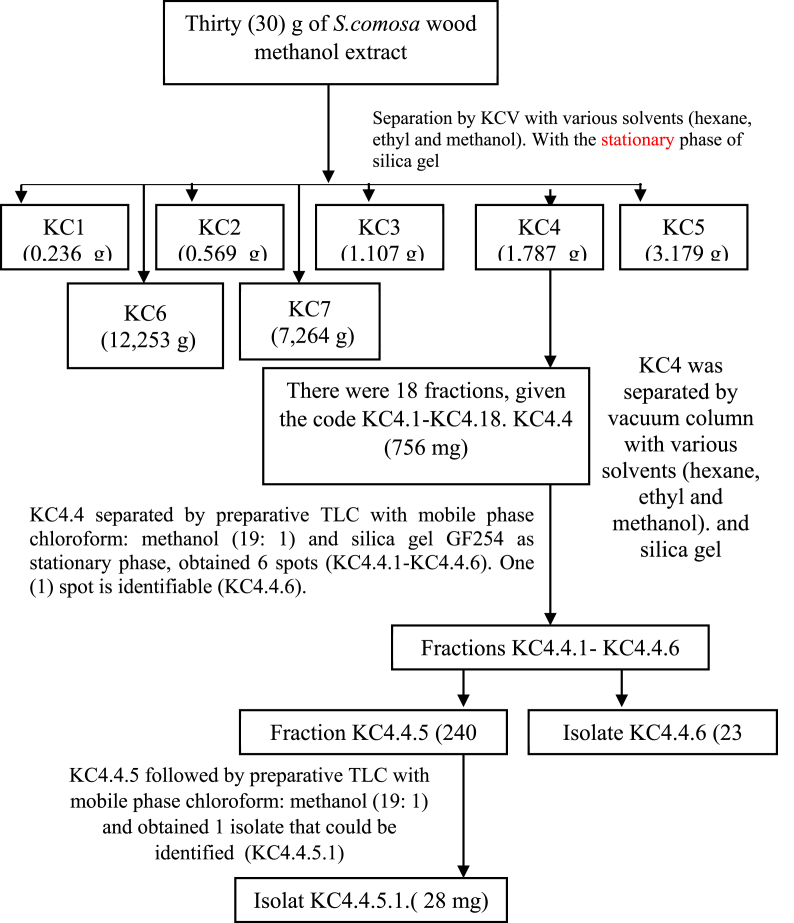
The isolation of chemical constituents from *Sterculia comosa*.

The isolation of chemical constituents from *Sterculia comosa* shown in Figure 1.

### 2.8 Molecular docking analysis

#### 2.8.1 Ligands preparation

Ligand structures were drawn by MarvinSketch 17.8. The 3d structure of each ligand was generated using VegaZZ, charge addition and energy minimization were done under the Vina force field in VegaZZ and saved in mol2 format.

#### 2.8.2 Macromolecule preparation

The 3D structure of arginase was obtained from RCSB with PDB id 4HZE (Zandt et al., 2013). The 3D structures of structural and non-structural protein of SARS-CoV-2 i.e. PLpro, 3CLpro, nsp12, and Spike obtained from RCSB with PDB id: 7CJM, 7JU7, 6NUS, and 6MOJ, respectively. Protein structure preparation done using AutoDock Tools. All non-standard residues and most water molecules were cleaned by removed from the initial structure except those involved in ligand-protein interaction. Then, all missing hydrogens and Kollman charges added to the system, and the prepared protein receptor then saved as pdbqt format (Ravi and Krishnan 2016).

#### 2.8.3 Docking validation

Docking validation was done through redocking of native ligand to its respective protein. The grid center was placed approximately to the ligand center, covering all the binding site residues. Grid box (40 × 40 × 40Å) centered at (34.891, 86.305, 71.497) Å for the arginase, grid box (40 × 40 × 40Å) centered at Å for the PLPro, grid box (40 × 40 × 40Å) centered at (13.449, 4.883, 22.106) Å for the 3CLPro, grid box (40 × 40 × 40Å) centered at (145.560, 154.865, 161.227) Å for the nsp12, and grid box (40 × 40 × 40Å) (−26.315, 35.602, 31.555) Å for the Spike. RMSD value of redocking and crystallography ligand must be less than 2Å to confirm the docking method's validity.

#### 2.8.4 Molecular docking analysis

All prepared ligands then were docked to arginase using Autodock 1.5.6. The valid docking parameters used to dock the test ligands against each protein. The docking parameters are specified as follows: GA runs 100, Pop size 200, ga_nu m_evals 2500000. Binding interactions between docked potent agents and the targets were analyzed using PLIP (Salentin et al., 2015).

## 3 Results

### 3.1 Arginase activity

**Figure 2:**
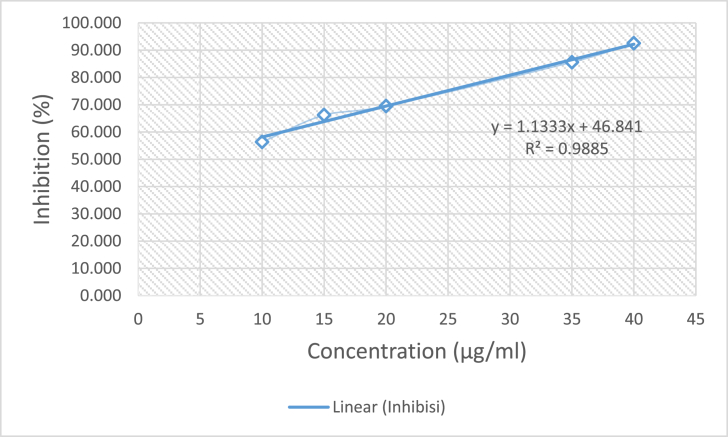
Arginase inhibitor activities of methanol extract of *S*. *comosa* woods.

The screening arginase activity showed that an active extract was methanol extract. The study of arginase activity performed on methanol extract obtained IC_50_ 2.787 μg/ml while the value of IC_50_ for nor-NOHA as a positive control was 3.773 μg/ml. The result shown in Table 1 the arginase inhibitor activity shown in Figure 2.

**Table 1:** Arginase inhibitor activity of extract methanol *S. comosa* (Wall) Roxb.

Concentration (μg/ml)	Average Inhibision (%)	kv	R^2^	IC_50_ (μg/ml)
10	56.344 ± 9.63	19.917		
15	66.221 ± 5.71	8.623	y = 1.1333 + 46.841	2.787
20	69.507 ± 10.98	14.710	R^2^ = 0.9885	
35	85.592 ± 6.89	8.052		
40	92.539 ± 4.04	4.263		
Nor-NOHA				3.733

### 3.2 Antioxidant activity

**Figure 3:**
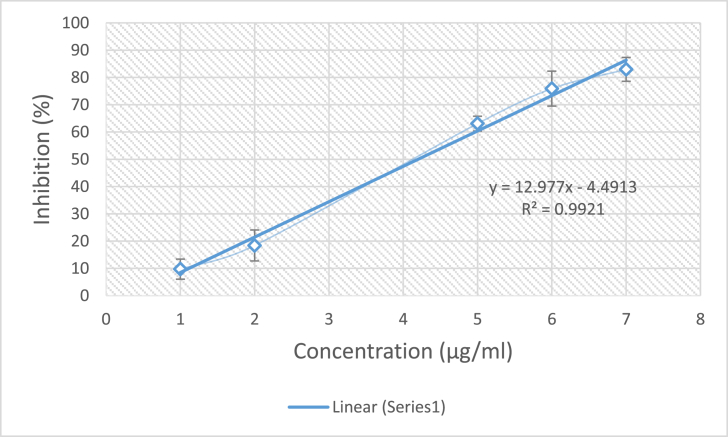
Antioxidant activity of methanol extract *S.comosa* woods with DPPH.

**Figure 4:**
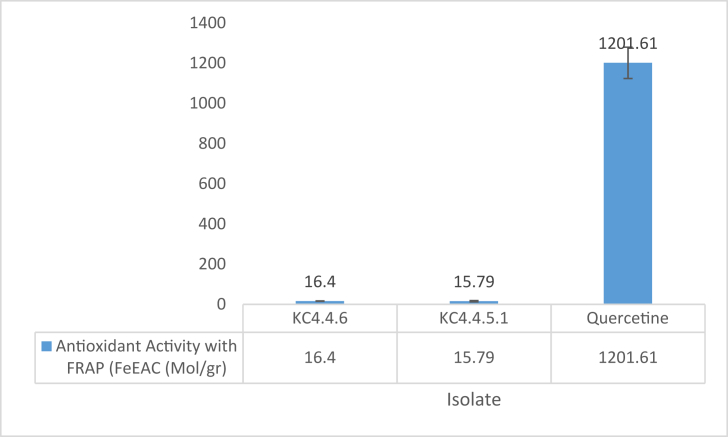
Antioxidant activity of isolate with the FRAP method.

The antioxidant activity using DPPH method obtained The IC_50_ 4,199 μg/ml. Quercetin, as a positive control, was 5.631 μg/ml. The result of the antioxidant activity in Table 2 and Figures 3 and 4. The FRAP method in Table 3. The antioxidant activity of extract with FRAP method 163,56 FeEAC (mol/g).

**Table 2:** Antioxidant activity of extract methanol *S. comosa* (Wall) Roxb. With DPPH.

Concentration (μg/ml)	Average Inhibision (%)	kv	R^2^	IC_50_ (μg/ml)
1	9.73 ± 3.68	37.831		
2	18.39 ± 5.67	30.834		
5	63.08 ± 2.71	4.297	y = 12.977x – 4.4913	4,199
6	75.91 ± 6.42	8.466	R^2^ = 0.9921	
7	82.96 ± 4.35	5.339		
Quercetine				5.631

**Table 3:** Antioxidant activity of extract methanol *S. comosa* (Wall) Roxb. With FRAP.

Sample	Antioxidant Activity FeEAC (mol/g)	SD	KV
*Sterculia comosa* Extract	163,56	2,61	1,59
Quercetine	1201,61	77,89	6,48

### 3.3 Determination of total flavonoids and total phenols

Quercetin levels calculated as total flavonoid levels in the sample. From the quercetin standard curve calculation, there is a linear correlation between absorbance and concentration with linear regression equation y = 0.0198x – 0.0215 and value of relation coefficient (R^2^) = 0.9964. Based on the measurement results, the average value of flavonoids in each gram of extract was 28.96 mg ± 3.74 QE/g. Gallic acids levels calculated as total phenols levels in the sample, with linear regression equation y = 0.026x + 0.3373 and value of relation coefficient (R^2^) = 0.9964. Based on the measurement results, the average value of total phenols in each gram of extract was 723.61 mg ± 54.72 GAE/g. Quercetin used as a standard for calculate flavonoids total, with linear regression equation y = 0.0198x – 0.0215 and value of relation coefficient (R^2^) = 0.9964. The result show in Table 4.

**Table 4:** Total flavonoids and total phenols in extract methanol *S. comosa*.

Extract	Total phenols (mg GAE/g)	Total flavonoids (mg QE/g)
*S. comosa*	723.61 ± 54.72	28.96 ± 3,74

### 3.4 Isolation

#### 3.4.1 Isolate KC4.4.6

**Figure 5:**
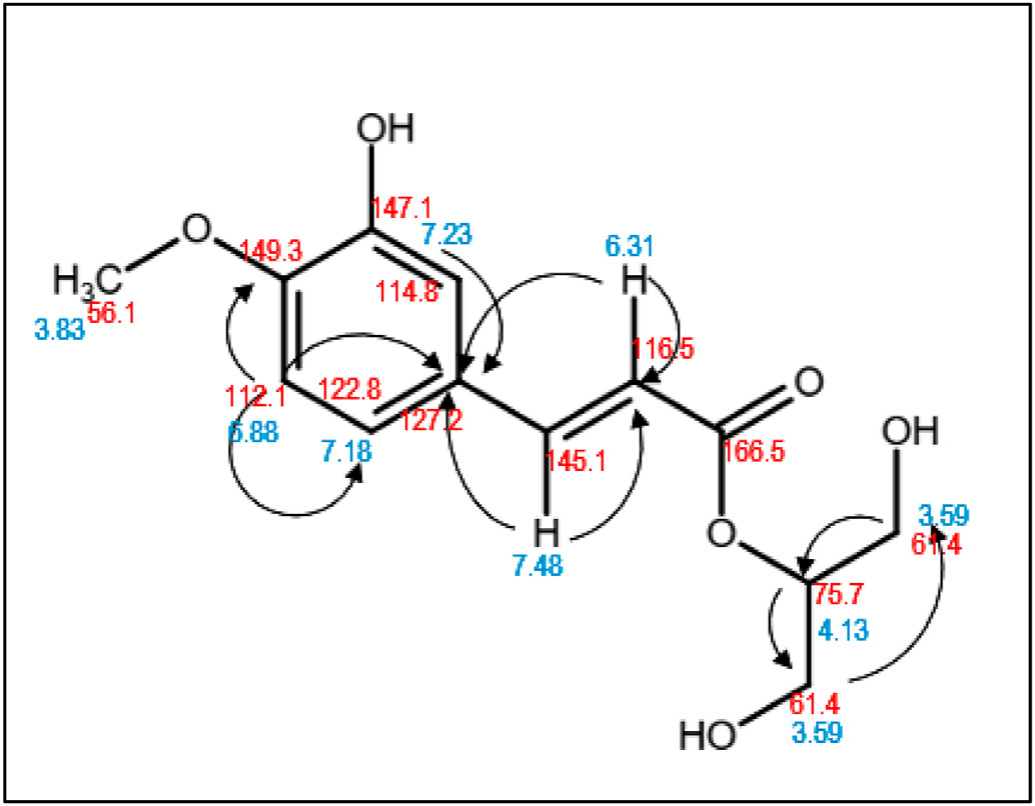
Isolate KC4.4.6 compound analysis with HMQC

Spectrum results show the presence of the main signal, namely the double bond in the trans form, which appears at 6.31 (d, 16 Hz) and 7.48 (d, 16 Hz), and the presence of methyl (s) appears at 3.83 (s). Besides that, the presence of an aromatic signal 3 appears at 6.88, 7.23, so this compound thought a cinnamic group. The results of the C-NMR measurement also reinforced this suspicion. The presence of –COO- groups appeared at 166.5 (s), 116.2 (d), and 145.1 (d). The HMBC results confirmed the allegations. So the provisional guess is (-)-2-(E)-caffeoyl-D-glyceric acid. This chemical contituent shows in Figure 5 and the result of chemical shift data from ^1^H-NMR and ^13^C-NMR spectra of KC4.4.6 compared with the literatur show in Table 5.

**Table 5:** Tabulation of chemical shift data from 1H-NMR and 13C-NMR spectra of KC4.4.6 isolates. Compared with reference.

		Isolate KC4.4.6		(-)-2-(E)-caffeoyl-D-glyceric acid	
		(500 MHz in CDCl_3_)		(225 MHz in CD_3_OD) (Hahn and Nahrstedt, 2002)	
		δ^13^C	δ^1^H	δ^13^C	δ^1^H
1	C	127,2	-	127,1	-
2	CH	114,8	7,23	113,6	7,07
3	C	147,1	-	144,6	-
4	C	149,3	-	147,7	-
5	CH	112,1	6,88	115,5	6,78 d (8,2)
6	CH	122,8	7,18	123,2	6,98 dd(2,1; 8,2)
7	CH	145,1	7,48	147,6	7,66 d(15,9)
8	CH	116,5	6,31	116,5	6,35 (15,9)
9	C	166,5	-	168,9	-
10	OCH3	56,1	3,83	-	-
1′	CH	75,7	4,13	172,6	-
2′	CH	61,4	3,59	74,1	5,16 dd (35; 4,7)
3′	CH	61,4	3,59	61,1	3,94 dd (4,01 dd)

KC4.4.6 shows a spectrum at 1765 cm-1 which indicates C = O bonds in the ester. The C = O group in the ester found at 1730-1750 cm-1 (Harmita, 2002). KC4.4.6 shows the presence of free O–H bonds shown at 3600 cm-1. According to the literature, free O–H found at 3600-3650 cm-1 (Harmita, 2002). KC4.4.5 (1) shows two groups (groups) of signals, namely the double bond in the transform appearing at 6.30 (d) and 7.30 (d) with a value of J = 16 Hz. The ABX system aromatic signals appeared at 6.72 (d, 8 Hz), 7.08 (d, 2.5 Hz), and 6.94 (s, 2.5; 8 Hz). The signal from the methoxy group (-OCH3) appeared at 3.85 (s, 3H). These results indicate that this compound is a trans-isoferulic acid.

Measurement data for the C-NMR spectrum support the estimated structure, as in the structure below, where the presence of a carboxylate group (-COOH) appears at 176.36. The correlation between H and C signals, like the results, is supported by HSQC correlation, as seen in the 2D HMQC spectrum. Signal 6.94 (dd) correlates with C at 122.84 ppm; signal H at 7.08 (d, 2.5 Hz) correlates with c at 111.19 ppm, and so on. The methoxy groups position strengthened by measuring the HMBC long-distance correlation, where the signal was 3.85 correlated with C at 149.38. Thus the position of the aromatic proton signal and the double bond is strengthened by the presence of HMBC correlation, it can be seen briefly in the structure and spectrum of Figure 5.

#### 3.4.2 Isolate KC4.4.5.1

**Figure 6:**
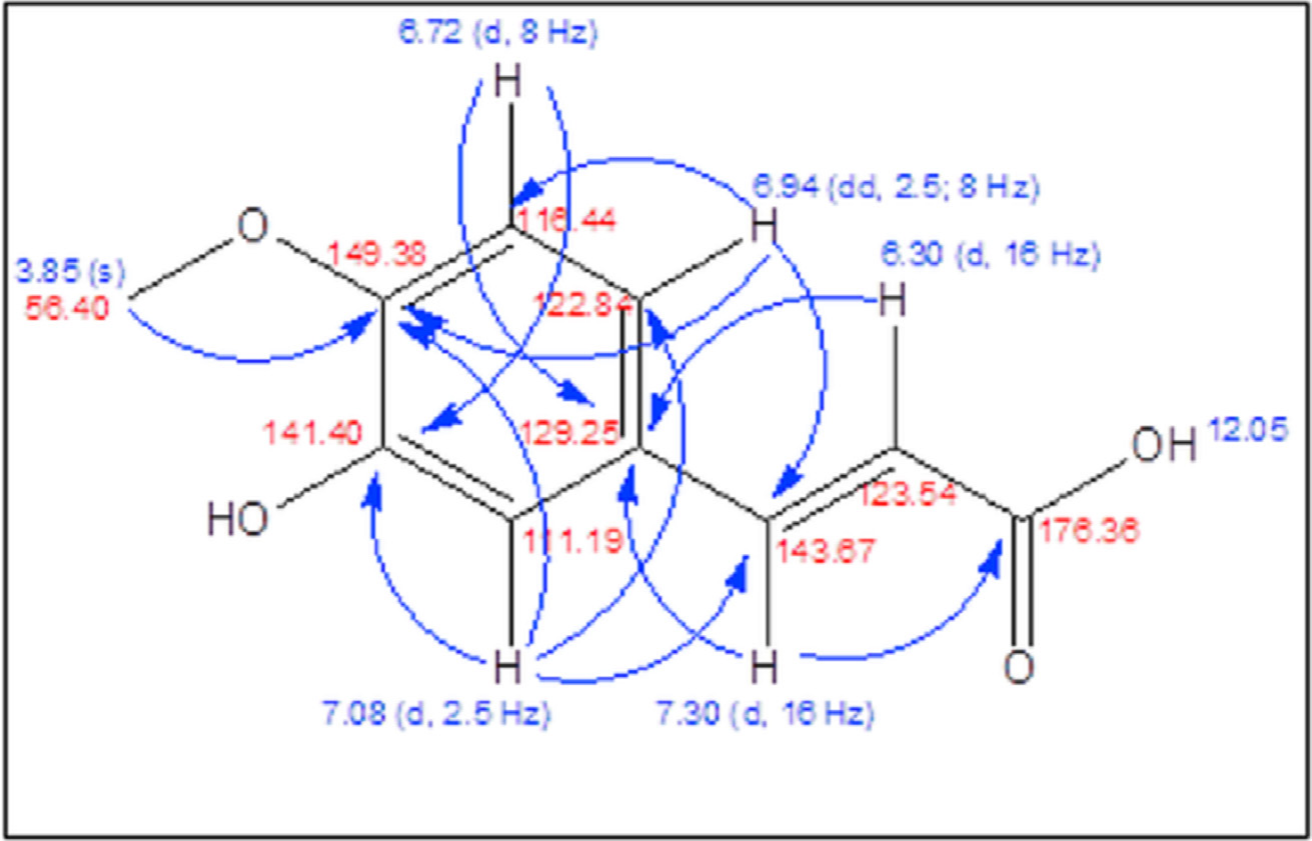
Chemical friction value of HMBC

This compound has a trans-isoferulic acid in the structure. The molecular formula C_10_H_10_O_4_ and molecular weight 194.0579. These results are consistent with the results of the LCMSMS. The IR spectrum of trans-isoferulic acid at 1738, 1687, 1670, 1631, 1599, 1510 cm^−1^ (Agarwal and Atalla, 2006). KC4.4.5.1 shows a spectrum at 1707 cm-1, indicating C = O. The C = O group in the carboxylate found at 1700-1725 cm-1 (Harmita, 2002). KC4.4.5.1 shows the presence of free O–H bonds shown at 3671, 3625 cm-1. According to the literature free O–H is found at 3600-3650 cm-1 (Harmita, 2002). Hydroxyl (O–H) in carboxylates is shown at 3091 cm-1 with moderate intensity, in the literature 2400–3400 (Harmita, 2002). At 1511 cm-1 which indicates the C = C bond in aromatic. The comparisons between NMR results and literature obtained the following data, listed in Table 6. The chemical structure show in Figure 6.

**Table 6:** Tabulation of chemical shift data of 1H-NMR and 13C-NMR spectra of KC4.4.5.1 isolates compared to the reference.

		Isolate KC4.4.5.1		Trans-isoferulic acid	
		(500 MHz in CDCL_3_)		(400 MHz in CD_3_OD + CDCl_3_) (Prachayasittikul et al., 2009)	
		δ^13^C	δ^1^H	δ^13^C	δ^1^H
1	C	129,54	-	126,83	-
2	CH	111,19	7,08 (d 2,5 Hz)	110,50,	7,07 (1H 1,67 Hz)
3	C	141,40	-	148,98	-
4	C	149,38	-	147,97	-
5	CH	116,44	6,73 (d 8,8 Hz)	115,57	6,87 8,00 Hz)
6	CH	122,84	6,91(dd 2.5; 8.8 Hz)	123,28	7,05 (1H, dd j = 8,00, 1,67 Hz)
7	CH	143,67	7,30 (d 16 Hz)	146,11	7,6 (d j = 15.70 Hz)
8	CH	123,54	6,30 (d 16 Hz)	115,28	6,26 (d, j = 15,70)
9	CO	176,36	-	178,25	-
10	OCH_3_	56,40	3,85 (s)	56,04	3,91 (3H, s)

### 3.5 The arginase inhibitor activity by isolates

Activity tests carried out on compounds that have been isolated show that the compound with the highest enzyme inhibitor activity is KM3.9.1 with an IC50 value of 59.31 μg/ml. NO is an essential molecule in regulating intercellular signaling and is involved in various processes, including regulating endothelial function. NO has antimicrobial activity, including against bacteria, protozoa, and some viruses. The result of Arginase Inhibitory Activity by Isolates show on Table 7.

**Table 7:** The arginase inhibitor activity by isolates.

	Isolat	IC_50_(μg/ml)		R^2^
1	KC4.4.6	98,03	Y = 0,3346x+17,198	0,8713
2	KC4.4.5.1	292,58	Y = 0,1392x+9,2732	0,9931
3	Nor-NOHA	3,97	Y = 5,4539x+28,378	0,9997

### 3.6 The antioxidant activity isolate

The antioxidant activity test results using the DPPH method showed that the compound with the highest antioxidant Activity was KC4.4.6 with an IC50 value of 48.77 μg/ml. The antioxidant activity test results using the DPPH method showed that the most active isolate was KC4.4.6. This compound found in *Sterculia comosa* wood. In the form of wood extract, *Sterculia comosa* has better Activity than *Sterculia macrophylla*. The presence of a hydroxyl group in the KC4.4.6 compound contributed to increased antioxidant and arginase inhibitory activity. In the pathological condition, when arginase activity increases, eNOS becomes uncoupling, uncoupling eNOS will produce more free radicals, namely O2- and ONOO-. The use of the DPPH method for which the radical scavenging mechanism will be suitable. So that free radicals produced from eNOs can be replaced by DPPH in this method of antioxidant activity. This result show in Table 8 and Figure 4 for FRAP antioxidant activity.

**Table 8:** The antioxidant activity with DPPH method by isolate.

Isolat	IC_50_(μg/ml)	Persamaan	R^2^
KC4.4.6	48,77	Y = 0,4764x+26,766	0,9922
KC4.4.5.1	88,08	Y = 0,1702x+18,497	0,9929
Quercetine	5,63	y = 8,0154x+4,8628	0,998

The antioxidant activity test results using the FRAP method showed high activity in the KC4.4.6 compound of 16.40 μg/ml. The results can show in Figure 4. These results are the same as the antioxidant activity test with the DPPH method, and the compound with the highest antioxidant activity is KC4.4.6.

The isolate results showed that the arginase inhibitory and antioxidant activity were smaller than the fraction, due to the possibility of synergism, complementary between compounds in the fraction that contribute to activity to increase the inhibitory activity of arginase and antioxidant enzymes.

### 3.7 Validation of docking method

**Figure 7:**
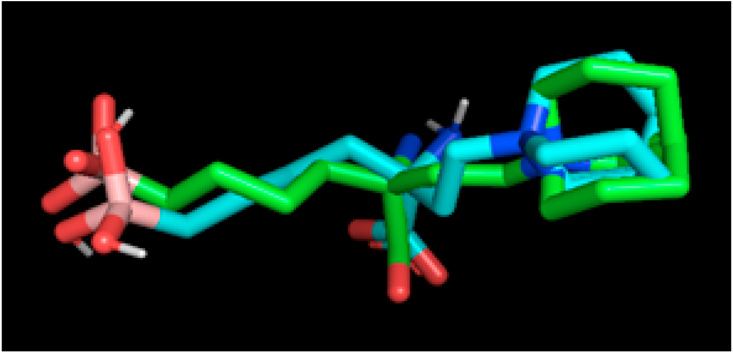
Overlay of redocked (blue) and crystallographic conformation (green) of the native ligand of arginase.

Validation of the docking method carried out on a complex of arginase with the native ligand. The result determine as the RMSD value, which states the difference in the distance between the atoms of the redocking native ligand and the X-ray crystallography results. The validation result showed that the RMSD was valued 1.342 Ǻ, so the docking method was valid. The overlay of redocked and crystallographic conformation of the native ligand can observed in Figure 7.

### 3.8 Docking results

Nitric oxide (NO) is an essential intercellular signaling molecule that inhibits some viral infections. NO reported to inhibit RNA synthesis and inhibit the SARS CoV replication cycle (Åkerström et al., 2005). Arginase is an enzyme that will break down arginine as the starting material for NO. Arginase inhibition will increase NO levels.

The native ligand of 4HZE, i.e. [(5R)-5-amino-5-carboxy-7-(piperidine-1-yl)heptyl] (trihydroxy)borate(1-), interacted to Asp147, Asn149, Ser156, His160, Gly161, Asp202 amino acid residues of 2-arginase by hydrogen bond. In contrast, non-hydrogen bond interactions observed to His120, His145, Asp200, as well as Asp202 (Zandt et al., 2013). Nor-NOHA actively inhibited 2-arginase and was used as a control compound (Tenu et al., 1999). Trans-isoferulic acid showed lower binding energy than native ligand and nor-NOHA. However, the binding energy of KC4.4.6 was higher than the native ligand (Table 8).

The results of molecular docking of the ligands against SARS-CoV-2 targets proteins are presented in Tables 9, 10, and 11. KC4.4.6. predicted have the best interaction with PLpro. However, both trans-isoferulic acid and KC4.4.6 did not show good interaction to 3CLPro, nsp12, and Spike (Table 10 and Table 11).

**Table 9:** Docking result to arginase (4HZE).

Compound	ΔG_binding_ (kcal/mol)	Amino acid residues involved in the interaction	
		Hydrogen Bond	Non-hydrogen Bond
Native ligand	-9.49	Asp147, Asn149, Ser156, His160, Gly161, Asp202	His120, His145, Asp200, Asp202
Nor-NOHA	-12.13	Ala146, **Asp147**, Asp200, **Asp202,** Glu205	**His120**, His160, A**sp200, Asp202**
Trans-isoferulic acid	**-14.72**	**Ser156**, Asn158**, His160**	**His120, His145**, His160
KC4.4.6	-6.41	**Asp147, Asn149,** Ser155, **Ser156, His160**	**His145**, His160, **Asp200**
Dihydroxy-dimethoxyflavone	-5.86	**Asn149**, Asn158, **Ser156, His160**, **Asp202,** Glu205	Thr265
Dihydroxy-trimethoxyflavone	-5.83	Gln37, Lys38, Arg39, **His160**, **Asp202**	Arg39
Piceatannol	-5.84	**Ser156,** Asn158, **His160**	Thr265
Isoquercetin	-6.71	**Asp147, Asn149,** Ser155, **Ser156,** Asn158, **His160,** Asp200, **Asp202,** Glu205, Gly264	Thr265

∗Bold font means the same interaction model with the native ligand.

**Table 10:** Docking Molecular Chemical Constituent with Protein target for COVID-19.

Chemical Constituent	Protein				
	4hze	4ow0 (PLpro)	7ju7 (3CLpro)	6nus (nsp12)	6moj (Spike)
KC4.4.5.1^a^	**-14.72**	-5.70	-4.30	**-6.21**	-5.30
KC4.4.6^a^	-6.41	-6.80	-4.95	-5.58	-5.22
Nat/ctrl∗	-9.49	-11.45	-8.76	-4.47 ∗	-7.95 ∗
NorNoHA^b^	-12.13	-7.20	-4.98	-3.85	-3.75
Remdesivir^c^	-5.67	-5.70	-4.91	-4.58	-5.43
Dihydroxy dimethoxyflavone^d^	-**5.86**	-6.85	-6.10	-5.55	-5.69
Dihydroxy trimethoxyiflavone^d^	-5.83	**-7.03**	**-6.22**	**-5.59**	-5.71
Piceatannol^a^	-5.84	-6.59	-5.67	-5.44	**-6.06**
Isoquercetine^d^	**-6.71**	**-7.05**	**-6.06**	**-6.29**	**-6.44**

Note:

∗: native ligand/control. Activity (in bold) is a compounds that have better activity than native ligands.

a chemical contituent of *Sterculia comosa*.

b positive control for arginase inhibitor.

c positive control for COVID-19.

d chemical constituent for arginase inhibitory activity.

**Table 11:** Docking Result Hyddrogen Interaction to Protein target for COVID-19.

Chemical Constituent	Protein				
	4hze	4ow0 (PLpro)	7ju7 (3CLpro)	6nus (nsp12)	6moj (Spike)
KC4.4.5.1^a^	SER156, HIS160	ASP165	CYS44, THR25, GLY143, **CYS145**	TYR456, SER682, **VAL560**	**ARG23**, PHE183, THR98
KC4.4.6^a^	SER155, HIS160, ASP147	ARG167, ASP165, TYR265	ASN142, THR26, HIS163	SER501, ASN507, GLN541, GLU665, LYS676, SER681, SER682, GLY683	**ARG23**,ASP96, THR98, PHE183
Nat/ctrl∗	ASP251, ASN149	TYR269	**CYS145**, HIS164	THR680, THR,540, VAL560, SER682, TYR456	ARG23
NorNoHA^b^	HIS160, ASP147, GLU205	**TYR269**, ASP165, LYS158, GLU168	CYS44, THR25, THR24	GLU665, **VAL560**, THR540, GLN541	PHE183, THR98, GLY99, ASP96
Remdesivir^c^	HIS160, HIS145, ASP200, SER155, ASP202	**TYR269**, GLU168	THR45, CYS44, GLU166, **CYS145**	SER682, **VAL560**, SER681, LYS676	**ARG23**, GLU184, LEU185, THR98, ASP96
Dihydroxy dimethoxyflavone^d^	HIS160, ASN158, **ASN149**, GLU205, HIS145	GLY267	GLU166, HIS41, GLY143	GLN541, GLU665, LYS676	GLU184, PHE183, THR98
Dihydroxy trimethoxyiflavone d	LYS38, GLN37, ASP202, HIS160	**TYR269**, ASP165	THR25, HIS41**, HIS164**, GLU166	GLN541, **VAL560**, SER681, GLU665	THR98, GLU184, **ARG23**, SER182
Piceatannol^a^	HIS160	LYS158	GLU166, GLY143, HIS41	LYS676, THR540, **VAL560**	THR98, LEU185, **ARG23**
Isoquercetine^d^	HIS160, ASN158, SER156, GLU205, ASP147, GLY161, ASP202, ASP200	**TYR269**, ASP165, TYR274	GLU166, HIS163, LEU141, GLY143, THR24,THR26,THR25,HIS41	SER681, GLU665, THR540, TYR456, **VAL560**, ASN507, SER501	PRO131, PHE183, GLU184

Note:

∗: native ligand/control. Amino acid (in bold) is amino acid that interacts with the native ligand.

a chemical contituent of *Sterculia comosa*.

b positive control for arginase inhibitor.

c positive control for COVID-19.

d chemical constituent for arginase inhibitory activity.

**Figure 8:**
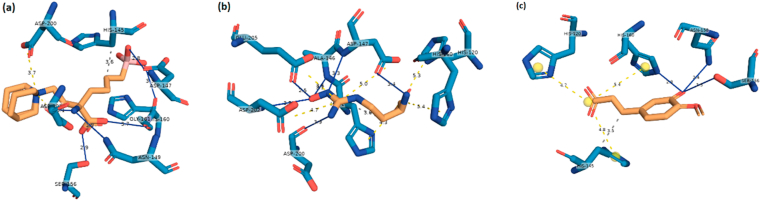
Interaction of native ligand (a), nor-NOHA (b), and isoferulic acid (c) to the amino acid residues of arginase.

The interaction of native ligand, nor-NOHA, and isoferulic acid to the amino acid residues of arginase can observed in Figure 8.

## 4 Discussion

Arginine metabolism plays an important role in vascular function. The enzyme associated with the metabolism of arginine was arginase. This enzyme also an effect on vascular function. Arginase and eNOS use L-arginine as a subtract. The competition of them give an affects on bioavailability NO, the decrease of NO will cause endothelial dysfunction (Olubukola Ayebimpe Akanni, solomon eduviere owumi, 2014). Flavonoids have antioxidant activity, and flavonoids can bind free radicals and donate hydrogen atoms or by electron transfer (Sofina Banjarnahor and Artanti, 2014). ONOO^−^ and H_2_O_2_ were oxidative species, they can increase arginase activity through PKC-mediated activation of RhoA/Rho kinase pathway. NO produced from endothelial cells activates guanylate cyclase in smooth muscle cells. It causes hyperpolarization and vasodilatation. Several studies have shown that reduced levels of NO contribute to vascular endothelial dysfunction (Chandra et al., 2012). Preliminary phytochemical analysis indicated presence of flavonoids, saponins, steroids and triterpenoids. The methanol extract of *Sterculia comosa* has the high of phenol content. Some of the compounds Flavonoids and phenols are widely studied have a large antioxidant activity. The compounds of phenol and flavonoids in this extract may contribute to the inhibitor of the arginase enzyme. Flavonoids are also responsible for the stimulation of antioxidant enzymes. Its ability to trigger the generation of antioxidant enzymes in human body. Flavonoids such as luteolin, fisetin can inhibit arginase enzyme (Sofina Banjarnahor and Artanti, 2014). Our study showed that *sterculia comosa* has an arginase inhibitory activity. This extract also have a high of total phenols and total flavonoids and this result as the same as our previous study. In the recent study the stem bark of *Caesalpinia turtuosa* has the arginase activity with the IC_50_ 11.58 μg/ml for methanol extract and 33.81 μg/ml for ethyl acetate extract; this result was the same as our study (Najid et al., 2018). a methanol extract of *Sterculia comosa* wood has the arginase inhibitory activity with IC_50_ value of 2,787 μg/ml and high phenol content of 723.61 mgGAE/g. It is possible for the phenol compounds contained in this extract to contribute to inhibiting arginase activity. This research was similar to the research by Oboh and Akamolafe, that high of phenol compounds include gallic acid, catechin, ellagic acid caffeic acid chlorogenic acid, and epicatechin contained in extracts with high arginase inhibitory activity (Akinyemi et al., 2016; Akomolafe et al., 2016).

NO produced by an enzyme that catalyzes L-arginine oxidation to NO and L-citrulline, namely NOS (Nitric Oxide Synthase). Aubrey-chimene et al. (2005) research results that NO specifically inhibits the SARS CoV replication cycle, especially at the beginning. NO production by iNOS produces antiviral effects (Aubrey-chimene et al., 2005). In the COVID-19 pandemic, neonatal patients' treatment with inhaled NO was beneficial (Lu, 2020). NO plays an important role in maintaining normal endothelial function. The reduced availability of NO causes cardiovascular, neurological, cancer, and respiratory disorders. The reduction in NO production occurs in line with the increased reactive oxygen species (Khaled et al., 2016). Based on Chandra et al. (2011), an increase in ONOO- and H2O2 oxidative stress will cause an increase in PKCα/β and then activate RhoA/Rho kinase (ROCK) and cause arginase activity to be excess. Based on this, the use of an antioxidant can function in inhibiting arginase activity.

Nor-NOHA as a control as an arginase inhibitor. The results of molecular docking to arginase revealed that Nor-NOHA formed 2 hydrogen bonds and 3 non-hydrogen bonds the same as the native ligand. Trans-isoferulic acid was the ligand with the lowest binding energy to arginase. It formed several identical hydrogen bonding and non-hydrogen bond interactions with the key active site amino acids compared to the native ligand. The phenolic group of iso-ferulic acid plays an important role in the interaction to Ser156, Asn158, and His160, as the hydrogen bond acceptor. The carboxylic acid of iso-ferulic acid act as a charge center to form electrostatic interaction to His120, His145, and His160 (Figure 2). KC.4.4.6 also formed some similar interactions as the native ligand. However, the higher energy binding probably due to the high distance of some of the hydrogen bonds. Dihydroxy-propyl substituent to carboxylic group of iso-ferulic acid reduced carboxylic acid interaction to the key amino acid residues, producing lower binding energy.

Native ligand (5-amino-2-methyl-N-[(1R)-1-naphthalen-1-ylethyl]benzamide) as PLPro inhibitor formed 3 hydrogen bonds with the amino acid residues of PLPro i.e. Asp 164, Tyr 268, and Gln 269. The isolate KC.4.4.6 and isoquercetin showed good interaction to PLPro binding site. This result was similar to the previous study, that quercetin glucoside had highest docking score, which binds to His74, Arg83, Tyr155, Asn157, His176 amino acid residues of viral protein at the binding pocket of PLpro (Hiremath et al., 2020). The docking score of remdesivir was lower that KC.4.4.6 and isoquercetin, due to remdesivir mechanism acted as antivirus by inhibiting RNA-dependent RNA polymerase (RdRp), not PLpro (Saha et al., 2020).

## 5 Conclusion

The chemical constituent of extract *Sterculia comosa* (Wall) Roxb has arginase inhibitory activity and antioxidant activity. The isolate trans-isoferulic acid predicted have good interaction to arginase. Isolate KC4.4.6. ((-)-2-(E)-caffeoyl-D-glyceric acid) was predicted to have good interaction to PLPro of SARS CoV-2 PLpro. However, both isolates did not show good interaction to 3CLPro, nsp12, and Spike protein of SARS CoV-2.

## Declarations

### Author contribution statement

Rini Prastiwi: Performed the experiments; Analyzed and interpreted data; Wrote the paper.

Muhammad Hanafi; Rani Sauriasari; Rina Herowati: Performed the experiments; Wrote the paper.

Yessi Desmiaty; Ema Dewanti: Performed the experiments.

Berna Elya: Analyzed and interpreted the data; Wrote the paper.

### Funding statement

This research supports The Ministry of Research, Technology, and Higher Education, The Republic of Indonesia, for the Research Foundation through the PDUPT Grant, number 96AD/LL3/PG/2020.

### Data availability statement

Data included in article/supplementary material/referenced in article.

### Declaration of interests statement

The authors declare no conflict of interest.

### Additional information

No additional information is available for this paper.
